# Anévrysme de l’aorte thoracique ascendante post traumatique probable: à propos d’un cas

**DOI:** 10.11604/pamj.2016.25.106.10722

**Published:** 2016-10-24

**Authors:** Abdellah Amine, Salah Belasri, Hicham Janah

**Affiliations:** 1 Cardiologie, 5^ème^ Hôpital Militaire Guelmim, Maroc; 2 Radiologie, 5^ème^ Hôpital Militaire Guelmim, Maroc; 3 Pneumologie, 5^ème^ Hôpital Militaire Guelmim, Maroc

**Keywords:** Aorte thoracique, anévrisme, traumatisme, Thoracic aortic, aneurysm, trauma

## Abstract

Les anévrismes de l'aorte thoracique représentent une affection relativement rare. Les étiologies sont dominées par l'athérosclérose, les dystrophies du tissu élastique et le traumatisme. Les auteurs rapportent le cas d'un patient âgé de 50 ans, ayant comme antécédents pathologiques un traumatisme thoracique sévère à l'âge de 25 ans lors d'un stage de parachutisme, qui consulte pour des signes neurosensoriels d'une hypertension artérielle. La radiographie pulmonaire montre une dilatation anévrismale de l'aorte thoracique. L'échocardiographie trans-thoracique, l'angio-scanner et l'imagerie par résonance magnétique objective un processus anévrismal de l'aorte ascendante. Le bilan d'extension et la recherche étiologique se révèlent négatifs. L'étude anatomo-pathologique de la pièce opératoire montre des lésions non spécifiques.

## Abstract (en)

Thoracic aortic aneurysms are relatively rare. Etiologies are dominated by atherosclerosis, elastic tissue dystrophies and trauma. We report the case of a 50-year old patient with a personal history of severe chest trauma occurring at age 25 years during parachute training. He presented with neurosensory signs of arterial hypertension. Chest X-ray showed aneurysmal dilatation of the thoracic aorta. Transthoracic echocardiography, CT angiography and MRI objectified ascending aortic aneurysm. The assessment of extent and etiological research were negative. The anatomopathological examination of surgical specimen showed nonspecific lesions.

## Introduction

L´anévrisme de l´aorte ascendante est retrouvé chez 3 à 4 % des patients de plus de 65 ans. Il est associé à un risque de complications létales important. L´enjeu est d´identifier les patients à risque afin de proposer un suivi et/ou une chirurgie prophylactique avant la survenue de ces complications.

## Patient et observation

**Figure 1:**
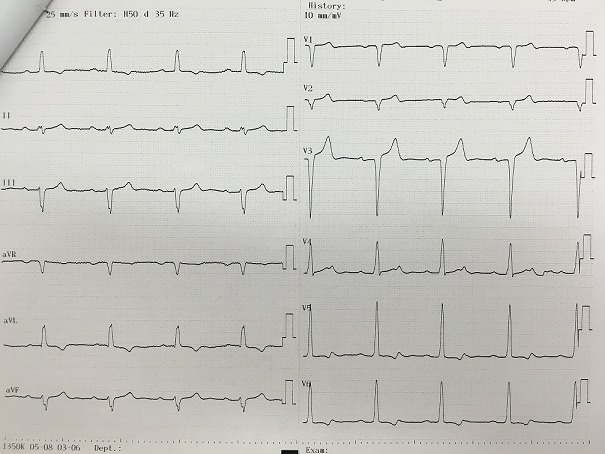
Electrocardiogramme montre bloc de branche gauche complet

**Figure 2:**
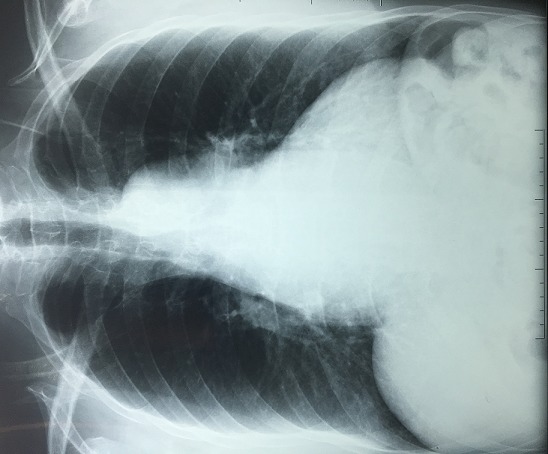
Radiographie pulmonaire de face montre un élargissement médiastinale

**Figure 3:**
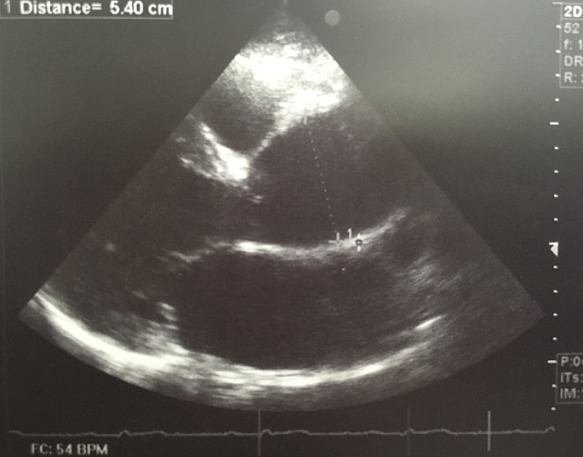
Echocardiographie trans-thoracique montre un anévrysme de l’aorte ascendante de 54 mm

**Figure 4:**
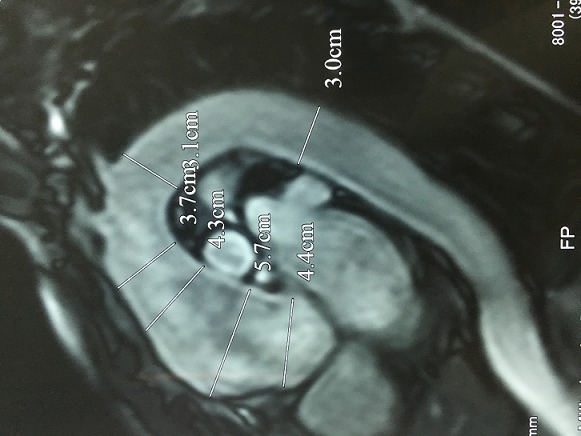
Angio-scanner thoracique montre un anévrysme fusiforme de l’aorte thoracique ascendante de 57 mm

**Figure 5:**
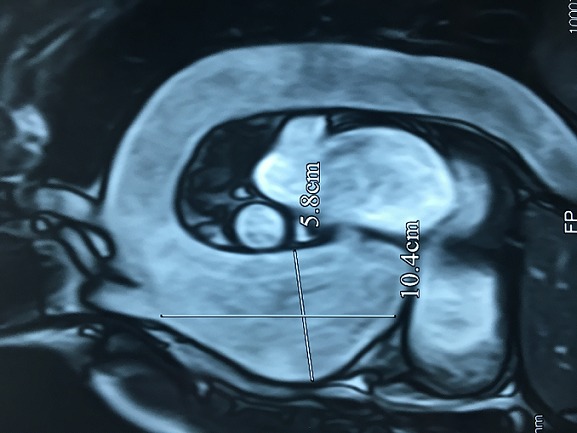
Angio IRM thoracique montre un anévrysme fusiforme de l’aorte thoracique ascendante de 58 mm

Il s'agit d'un homme âgé de 50 ans sportif qui consulte pour une hypertension artérielle (HTA) de découverte récente avec une dyspnée d'effort stade II de la NYHA d'installation progressive accompagnée de signes neurosensoriels d'HTA. Dans ces antécédents on retrouve une notion de chute avec point d'impact thoracique violant lors d'une compétition de parachutisme à l'âge de 25 ans. L'examen clinique trouve un patient en bon état général, la tension artérielle aux deux bras est à 180/90 mmHg, l'auscultation cardiaque retrouve un souffle diastolique au foyer aortique. Les pouls sont présents et symétriques. Il n'y a pas de signe clinique d'insuffisance cardiaque. L'électrocardiogramme enregistre un bloc de branche gauche complet (Figure 1). La radiographie pulmonaire de face montre un élargissement médiastinale (Figure 2). L'échocardiographie trans-thoracique montre un anévrysme de l'aorte ascendante de 54 mm de diamètre (Figure 3) avec un ventricule gauche dilaté et hypokinésie globale, une fraction d'éjection systolique du ventricule gauche est estimée à 40%. Au doppler on retrouve une insuffisance aortique importante. L'angio-scanner thoracique montre un anévrysme fusiforme de l'aorte thoracique ascendante de 57 mm (Figure 4). Un angio IRM thoracique affirme les mesures d'un anévrysme fusiforme de l'aorte thoracique ascendante de 58 mm (Figure 5). Une coronarographie est réalisée montrant un réseau coronaire angiographiquememt sain. Le reste du bilan biologique et radiologique préopératoire et étiologique est normal. L´intervention chirurgicale a consisté en un remplacement par prothèse mécanique de la valve aortique et de la racine de l'aorte (procédure de Bentall) sous circulation extracorporelle. L´évolution postopératoire était satisfaisante. L´examen microscopique de la pièce opératoire était en faveur de lésions non spécifiques. L´hypothèse post-traumatique ancienne est évoquée.

## Discussion

La physiopathologie des lésions traumatiques de l'aorte thoracique est complexe. La fréquence élevée des lésions de l'isthme est en partie liée à sa position anatomique, relativement fixe au sein de la cage thoracique [1]. Lors d'un traumatisme à haute cinétique, tel que peut l'être un accident de la voie publique, l'aorte thoracique subit une forte décélération associée à une compression de la paroi thoracique. S'y associent des lésions complexes de distorsion et d'étirement de la paroi aortique, particulièrement aux points d'ancrage, expliquant ainsi la fréquence élevée des lésions de l'isthme aortique [1, 2]. À l'inverse, les lésions de l'aorte thoracique ascendante sont probablement aussi fréquentes, mais s'observent moins souvent en raison de leur gravité, responsable de décès immédiat fréquent [1, 3]. Les circonstances de survenue sont par ordre de fréquence décroissant : les accidents de voie publique, les accidents d'écrasement, les défenestrations et les autres accidents de décélération lors de pratique sportive telle que le ski [2, 4, 5]. La plupart des anévrysmes de l'aorte ascendante restent asymptomatiques pendant une longue période. Le diagnostic est souvent fait sur une radiographie de thorax ou d'autres examens d'imagerie. L'examen clinique est peu contributeur. Un anévrysme est souvent associé à une insuffisance aortique avec un souffle diastolique, ou, des symptômes d'insuffisance cardiaque. Un examen approfondi vasculaire doit être effectué pour chercher une maladie vasculaire périphérique concomitante, une maladie carotide, ou des séquelles d'embolisation périphérique. Les signes cliniques chez le patient avec anévrysme de la racine aortique varient en fonction de la cause. Les patients atteints du syndrome de Marfan ont des caractéristiques squelettiques. Il est essentiel chez les patients ayant un anévrysme de l'aorte thoracique d'évaluer les autres membres de la famille. Le facteur essentiel dans la décision opératoire est l'évolutivité du diamètre de l'aorte, imposant plusieurs contrôles par l'imagerie pour mettre en évidence, dans un délai déterminé, une augmentation substantielle de ce diamètre, seuls les anévrysmes vraiment volumineux ou entrainent des complications conduisent à une chirurgie sans délai, prenant en compte l'histoire naturelle des anévrysmes de l'aorte thoracique ascendante, la chirurgie semble appropriée lorsque le diamètre maximal atteint 50-55 mm en fonction de l'étiologie. Dans notre cas clinique, la chirurgie était indiquée en présence d'un anévrysme de l'aorte thoracique ascendante supérieur à 55mm associée à une insuffisance aortique importante.

## Conclusion

Une évolution spectaculaire est observée en technique de prise en charge des anévrysmes de l'aorte thoracique et de nouvelles perspectives se sont ouvertes depuis plusieurs années. Néanmoins il s'agit toujours d'une pathologie potentiellement mortelle. Ceci dit l'intérêt du dépistage des patients à risque afin de réduire au maximum la survenue de cette issue dramatique.
